# Infectious disease and health systems modelling for local decision making to control neglected tropical diseases

**DOI:** 10.1186/1753-6561-9-S10-S6

**Published:** 2015-12-18

**Authors:** T Deirdre Hollingsworth, Ivor Langley, D James Nokes, Eleanor E Macpherson, Gerry McGivern, Emily R Adams, Moses J Bockarie, Kevin Mortimer, Lisa J Reimer, Bertie Squire, Stephen J Torr, Graham F Medley

**Affiliations:** 1 University of Warwick, Coventry, UK; 2 Liverpool School of Tropical Medicine, Liverpool, UK; 3 KEMRI-Wellcome Trust Research Programme, Kilifi, Kenya; 4 London School of Hygiene and Tropical Medicine, UK

## Abstract

Most neglected tropical diseases (NTDs) have complex life cycles and are challenging to control. The “2020 goals” of control and elimination as a public health programme for a number of NTDs are the subject of significant international efforts and investments. Beyond 2020 there will be a drive to maintain these gains and to push for true local elimination of transmission. However, these diseases are affected by variations in vectors, human demography, access to water and sanitation, access to interventions and local health systems. We therefore argue that there will be a need to develop local quantitative expertise to support elimination efforts. If available now, quantitative analyses would provide updated estimates of the burden of disease, assist in the design of locally appropriate control programmes, estimate the effectiveness of current interventions and support ‘real-time’ updates to local operations. Such quantitative tools are increasingly available at an international scale for NTDs, but are rarely tailored to local scenarios. Localised expertise not only provides an opportunity for more relevant analyses, but also has a greater chance of developing positive feedback between data collection and analysis by demonstrating the value of data. This is essential as rational program design relies on good quality data collection. It is also likely that if such infrastructure is provided for NTDs there will be an additional impact on the health system more broadly. Locally tailored quantitative analyses can help achieve sustainable and effective control of NTDs, but also underpin the development of local health care systems.

## Background

Neglected tropical diseases (NTDs) are caused by pathogens that have successfully overcome the biological challenges of persistence in the human population (especially host immunity), but have previously failed to raise a political profile that would generate an effective control programme (unlike smallpox, HIV etc.). Endemic diseases typically have a very low profile: TB is perhaps the only exception of a highly endemic disease with a high political profile and therefore substantial research funding. Almost all NTDs have at least one of a selection of features that make them hard to control, either by host immunity, or by public health intervention:

• Poor diagnosis

∘ Resulting in a large proportion of infections which are clinically indistinct (high asymptomatic prevalence)

∘ Poor immunological markers (mirroring the relative ineffectiveness of host immunity)

• Ineffective vaccination

∘ Mirroring the relative ineffectiveness of host immunity

• Regional high prevalence

∘ Targeted treatment not feasible or unlikely to be successful

∘ Asymptomatic infections contribute most to transmission, creating a rift between clinical management (which concentrates on the most sick) and public health (which should concentrate on the “infectious well”).

• Maintenance of very low prevalence with spatially concentrated foci

∘ Mechanisms for maintenance may be unclear (e.g. Human African Trypanosomiasis, HAT), perhaps mediated by reservoir hosts or long infectious periods in asymptomatic hosts

∘ Challenging to break the transmission cycle

• Non-human reservoirs and / or environmental contamination

∘ Provides a target for control, but often compromised by ubiquity of environmental contamination / vector density

• Long incubation period and / or low apparent pathogenicity (i.e. chronic disease rather than acute death)

∘ Low political profile and interacts with diagnostic problems. These are also features which are highlighted in the chronic and non-communicable disease discussion papers.

• Transmitted via ubiquitous behaviour

∘ Typically the risk behaviours are essentials (e.g. washing, sleeping, eating)

• Typically diseases of poor, marginal, populations with low political profile

∘ This is circular and self-reinforcing in that these people are compromised by the burden of disease that they bear.

These characteristics are not solely the characteristics of the core NTDs, but also other infections which are not classified as NTDs such as, arguably, *Plasmodium vivax* and *Plasmodium ovale*. NTDs are essentially endemic diseases in host populations with weak infrastructure (e.g. sewerage, water purification). Of course, transmission of several NTDs can be curtailed by improvements in housing, infrastructure and health systems, as was the case for soil-transmitted helminths in the USA[1] and South Korea[2]. It is only since the 1993 World Bank report[3] that it has been commonly accepted that one of the reasons for poverty and low infrastructure is the burden of disease, thus breaking circularity and allowing NTDs to develop a political profile. It is now generally accepted that controlling NTDs will facilitate economic development that will then enable the infrastructure.

Control of NTDs in the absence of massive development of infrastructure and healthcare facilities, requires multiple approaches in integrated targeted control programmes. Technological solutions, or ‘magic bullets’ such as a near perfect diagnostic or vaccines, can attract funding and resources, whereas creating broad support for complex and ‘messy’ solutions from funding agencies, politicians and affected populations is more challenging. Legal frameworks and popular perception mean that the efficacy of individual treatments have to be high, so that vaccines that provide 30% protection are unlikely to be licenced or perceived to be successful. However, if they were combined with a behavioural intervention and a targeted treatment programme, they might be an essential element of a highly successful integrated intervention.

This situation leads to the need to develop local initiatives within which programme managers can develop combination interventions that are consistent with local behaviours and are culturally acceptable. Examples of low tech interventions are straining of drinking water (against dracunculiasis) and insecticide treated bed-nets. Mixing these interventions with higher tech interventions (such as spraying insecticides, or mass treatment), can be highly successful, but the details of the culturally acceptable / appropriate operational aspects have to be considered in terms of the public health impact. For example, delivery of mass anthelmintics to school children has to be timed with the school year, which might or might not interact most appropriately with seasonal transmission. Similarly, insistence on collecting three sputum specimens to define sputum smear positive was a global tuberculosis (TB) control requirement but was of questionable value. Thus, it failed to promote access to services and in countries where poor people pay substantial out-of-pocket expenditures to access health facilities it increased those costs (see CAHRD Paper LH Costs).

In lower-resource settings efficient allocation of resources is essential and therefore requires that interventions have optimal effectiveness within local constraints. This will vary considerably at a sub-national scale, for example between rural and urban areas[4] and therefore international guidelines are unlikely to be flexible enough to give advice on appropriate interventions for every setting.

## Need for quantitative analysis and modelling

This need for locally applicable solutions using complex combinations of interventions requires the assistance of modelling in policy design[5-7]. Modelling has been used highly successfully in “command and control” national programmes in developed countries to design control programmes against childhood viral infections (MMR) and pandemic influenza[5,8,9]. Modelling has also been used at national and international scales to plan for pandemic influenza and advocate for interventions against HIV[10,11]. In a number of high resource countries it would now be unacceptable for a vaccination programme to be proposed for which the appropriate modelling had not been performed to demonstrate both effectiveness and cost-effectiveness[12,13]. In the UK, the Joint Committee on Vaccination and Immunisation (JCVI) has three sentences as its terms of reference, and the first is: “To advise UK health departments on immunisations for the prevention of infections and/or disease following due consideration of the evidence on the burden of disease, on vaccine safety and efficacy and on the impact and cost effectiveness of immunisation strategies”[14]. Its most recent decision on the introduction of a meningitis B vaccine (2014) is based almost entirely on the cost-effectiveness of the intervention as predicted by mathematical modelling[15].

Despite the demonstrable usefulness of mathematical models and quantitative analysis in informing policy, globally only a small number of countries have the necessary frameworks and capacity within which modelling and policy have developed strong, close relationships that have enabled health policy to be guided by quantitative models. Clearly, it is not a simple process to build these relationships, and therefore there is a need to consider how these tools and expertise can be developed and used in an effective way to support the control and elimination of NTDs.

Models are best developed and used in collaboration with the policy makers and practitioners – to ensure they are addressing the right questions, making the best use of all available data and to make sure that the assumptions and uncertainties in the model are properly communicated[16]. Further, policy makers need support in becoming intelligent users of models and quantitative evidence. An additional benefit of bringing modelling closer to policy and practice is to demonstrate the value of data collection activities, or of including some questions in routine surveillance. For example, during the SARS outbreak in 2003, mathematical epidemiologists were involved in the design of data collection activities in Hong Kong, leading to more powerful analyses[17].

For NTDs the need for these models goes beyond the current 2020 targets[18,19] to long-term control and true local elimination of transmission, with countries being increasingly expected to take on the financial costs (see CAHRD Paper NTD Delivery). There are currently a whole range of transmission models and novel statistical analyses being developed (NTD Modelling Consortium [20], http://www.parasitesandvectors.com/series/ntdmodels2015, led by the University of Warwick). There is a marked lack of health systems and cost-effectiveness modelling in NTD control[21]. The question which is posed in this discussion paper is how to bring these tools and insights through to local policy, including extending from transmission modelling to health systems and cost-effectiveness modelling.

## Bringing quantitative tools to local decision making

The vision for this discussion paper is that we should work towards providing mathematical and statistical tools that can be used to inform and support highly effective, locally tailored interventions for these highly spatially heterogeneous infections. To be effective these analyses need to arise as part of an effective co-operation and collaboration between local stakeholders – community representatives, healthcare workers, (national) politicians and epidemiologists (modellers). It requires modelling teams working locally (sub-nationally), but located within regional centres of excellence, ideally tied to local laboratory (surveillance) capacity. This also requires that modellers overcome hurdles associated with communicating quantitative sciences to non-specialists, an issue which affects many aspects of public health, not just NTDs.

More local modelling has to fit with the roles of local authorities and community leaders., as well as integrating with current frameworks. All the components of health systems will move in one way or another once any change is effected on any of the building blocks (human resources, information, financing, governance, service delivery, etc.). Any move to create local capacity for NTD modelling should aim to strengthen other health system components. As data analysis and synthesising and modelling are generally useful to health care evaluation and planning, such integration is likely to prove beneficial. To some extent, the structure of such as system would depend on how NTD is situate within the organisation.

This network would not be addressing a philosophical or theoretical need. Programme managers are seeking to design the best intervention for their population and regularly ask for guidance on how they should design complex interventions in *their* situation. For example

• How well is my intervention working?

• How much longer will I need to hold it in place?

• What combination of interventions should I be using where?

• What resources and costs will I need to secure now and in the future?

• What synergies are available between different interventions?

• What are the opportunity costs of different interventions?

• What is the impact on patient access and costs?

• How large is the undiagnosed epidemic?

Of course, although data analysis/synthesis and modelling can address the effectiveness aspects of these questions, economic evaluation is required to address the cost aspects. This is a major undertaking in itself, and much of what we have written about modelling could also be written about economic evaluation: it is context sensitive and under-resourced, so that supra-national results are having to be used to make local decisions (for example,[22]).

Programme managers look to the World Health Organization (WHO) and other international bodies for guidance, but often there is a need to tailor to local need. For example, the decision of whether to introduce an expensive diagnostic depends on local cost-effectiveness[23]. Local capacity would also address problems of analysis being done far from the point of need, the need to keep data and analysis close to the populations to which they apply and for the analysis to be an active discussion targeted at improving the local intervention rather than for academic impact. Modellers would also need to have a clear understanding of local realities, rather than be led by pre-conceived ideas of how processes work.

The key to modelling, whatever the scale, is that it is based on accurate data. Data are always collected locally, although frequently then collated at a larger scale with much of the information potentially lost. A likely major advantage of moving modelling towards the point of data collection is that the perceived value of the data increases, which consequently drives up quality, completeness and timeliness, benefitting all levels of analysis.

Another key argument for local modelling is that NTDs are highly dependent on local ecologies and behaviours. Consequently, as national and regional control of NTDs is achieved, there will be “hot spots” of infection (as has been seen for malaria[24]) for which general interventions are insufficient or inappropriate. Following control and then local elimination, intervention programs maybe halted, but on-going well-designed surveillance and monitoring will be required to maintain these gains. Ultimately all NTD elimination will be local, so developing a local capacity now is both providing the opportunity to improve the design of current interventions and improving preparation for the “final mile” and true elimination. This requires not only good transmission modelling, but also better models of health systems. If this can be done for NTDs it can also strengthen capacity and health systems with positive effects for public health more generally.

## The LSTM/Warwick collaboration

The collaboration between LSTM and the University of Warwick provides a unique opportunity to develop this vision. Warwick Infectious Disease Epidemiology Research (WIDER) is an internationally renowned centre for infectious disease modelling, with experience in training modellers through undergraduate, graduate and extra-mural courses. The team at Warwick has considerable experience in training modellers and can provide an international resource and underpin local capacity in development of modelling frameworks. LSTM and its global partners have the international experience in supporting capacity strengthening (see CAHRD Paper HS Capacity) and in delivering interventions to improve health systems. LSTM has played a role throughout the pathway from basic research through to new tools, which lead to new strategies. They have experience in NTD data collection and intervention evaluation, but more importantly the expertise to provide the experience of cultural sensitivity to design management systems required to adapt interventions to local settings. Local interventions should be designed by locals, but this requires a framework and structures that are able to combine the hard sciences of modelling and data analysis with the softer sciences of sociological, economic and political management. Such integrated tools support:

1. Planning policy and predicting impact of interventions.

2. Estimating current state, interpreting surveillance data and predicting effectiveness of interventions.

3. Identifying the most effective way to roll-out interventions.

4. Calculating cost-effectiveness of interventions within local settings.

## Issues to be discussed

There are a number of questions which need to be addressed in providing these tools and capacity. They include, but are not limited to, the following:

## A Technical questions

1. What level of expert knowledge should be required to run the modelling tools and statistical analyses? Should there be an automatic interaction with decision management tools, maps etc.

This will depend on the functionality required and the disease being modelled. An automated tool has many benefits in terms of rapidity of results and analyses and ease of use and integration with decision support tools (see CAHRD Paper NTD Tools). There are risks associated with fully automated tools, however, including loss of faith in the results when incorrectly used or interpreted and risks of the “wrong” model being used if not done automatically. Therefore, even with automated tools, local expertise has to be sufficiently expert. The choice between fully automated and more “hands-on” tools will be a delicate balance between giving the tools to the local decision makers and making sure quality is maintained. It is therefore important to involve both modellers and stakeholders in the design of any interfaces.

2. Should one model or multiple models be used at the programmatic level?

As recognised with the NTD Modelling Consortium, there is a need for multiple models scientifically and at strategic level, but are they needed at the programmatic level? Using multiple models can create a false sense of security if they are actually very similar models and it can be difficult for a non-expert to interpret differences between models. But it may be important to highlight these differences. Perhaps it would be possible to develop a new approach to determining which of a suite of models is most appropriate for different circumstances. At present there can be argument about which is the “best” model – but the real question is which is the most “useful” model – or even better, which is the most “useful result”. It is important to remember that modelling is only a means to an end: use of the modelling results, and their impact on policy and interventions, are the end points.

3. How local is local?

There is clearly an epidemiological / ecological spatial scale for these diseases, but there are also political and social scales, which frequently do not overlap neatly - national and regional boundaries were not designed to make infectious disease control easy. Therefore the level at which these models need to be used will depend on the disease and location for which they are being developed. This question also addresses how human expertise should be distributed, from international to local levels: is there sufficient resource for a talented modeller at each location, or should modellers be national, with biometricians more locally?

## B Finding the right mechanisms

4. What does previous experience tell us?

There are two major centres for infectious disease modelling in middle-income countries. The first is the South African Centre for Epidemiological Modelling and Analysis (SACEMA), which provides epidemiological modelling analyses for a number of African countries, with a particular strength in HIV and tsetse-borne trypanosomiasis. The second is the Mathematical and Epidemiological Modelling unit (MAEMOD) at the Mahidol-Oxford Tropical Medicine Research Unit in Bangkok, Thailand. Both have many years of applying epidemiological modelling to low-resource countries and building regional capacity.

5. What is the right mechanism for generating demand, developing capacity, supporting capacity?

This depends on what level of skills are required, whether it is short-term training courses through to doctoral or post-doctoral training and exchanges. It also depends on the extent to which different levels of local policy makers need to be informed about the interpretation and use of models. There is also a need to frame the capacity development in terms of overall capacity strengthening, with explicit goals and processes (see CAHRD Paper HS Capacity).

6. What should we prioritise?

Commonalities between different modelling approaches and methodologies should mean that by building the models which policy-makers need for one disease and training people on one area of modelling will impact others, both within and outside NTDs (see CAHRD Paper HS Capacity). Depending on local need, or availability of data, human capacity or other resources, LSTM/Warwick may be able to assist in different areas in different countries. In some countries it may be best to prioritise those NTDs for which mass drug administration planning is urgently required, whilst in others it may be best to start with outbreak analysis for diseases such as HAT.

## Summary

The 2020 goals for NTD are highly laudable and will deliver huge public health impacts across the NTDs[18,19]. Models are an essential tool in planning policy and estimating the resources required and the timescales over which targets can be achieved. However, over the 10-20 year timescale, breaking transmission cycles for some of these diseases is going to be extremely challenging, and true elimination may require special measures, and might even be beyond current technology and competence (see CAHRD Paper NTD Tools). Control and elimination will require complex combinations of interventions tailored to local need and social context, as highlighted in the other discussion papers (see CAHRD Papers NTD Tools and Delivery), and to the local social context. Quantitative analyses will be an essential part of adapting these programmes in the most effective way - the analyses and modelling that have become standard for developed countries should be made available more widely. Developing this capacity will also have impact on health systems more broadly, enabling local health systems to add an additional skillset to improve health outcomes. Investment in NTD control and elimination programs, aligned with a general strengthening of health systems, will leave a valuable legacy that goes beyond the primary aim of controlling these diseases.

## Competing interests

The authors declare no competing interests.

## Authors' contributions

TDH, BS, ST and GFM conceived the study. All authors contributed to discussions of the content and drafting the manuscript.

## Declarations

This article has been published as part of *BMC Proceedings* Volume 9 Supplement 10, 2015: The 2014 CAHRD (Collaboration for Applied Health Research & Delivery) Consultation. The full contents of the supplement are available online at http://www.biomedcentral.com/bmcproc/supplements/9/S10.
